# The CAMPUS Study: A Systems Approach to Alcohol-Involved Sexual Violence on College Campuses

**DOI:** 10.15288/jsad.24-00016

**Published:** 2025-03-31

**Authors:** Christina F. Mair, Michelle Dougherty, Travis R. Moore, Robert W. S. Coulter, Jessica G. Burke, Elizabeth Miller

**Affiliations:** ^a^ Department of Behavioral and Community Health Sciences, School of Public Health, University of Pittsburgh, Pittsburgh, Pennsylvania; ^b^ Center for Social Dynamics and Community Health, School of Public Health, University of Pittsburgh, Pittsburgh, Pennsylvania; ^c^ Department of Epidemiology, School of Public Health, University of Pittsburgh, Pittsburgh, Pennsylvania; ^d^ Department of Nutrition Interventions, Communication, and Behavior Change, Friedman School of Nutrition Science and Policy, Tufts University, Boston, Massachusetts; ^e^ Department of Community Health, School of Arts and Sciences, Tufts University, Medford, Massachusetts; ^f^ Department of Pediatrics, University of Pittsburgh School of Medicine, Pittsburgh, Pennsylvania; ^g^ Division of Adolescent and Young Adult Medicine, UPMC Children's Hospital of Pittsburgh, Pennsylvania

## Abstract

**Objective:**

Developing a better mechanistic and multilevel understanding of sexual violence on college campuses can help us evaluate and implement existing interventions, as well as develop new ones. We brought together scientists, practitioners, and college students to collaboratively characterize the systems surrounding alcohol-involved sexual violence on college campuses. Using collaborative model-building, they created models that highlight interconnected and multilevel influences and consequences of sexual violence.

**Method:**

Collaborative model-building activities involved two collaborator groups (12 students and 8 practitioners) and a core modeling team (7 scientists). Each collaborator group met for four 2-hour sessions to develop systems models of alcohol use and sexual violence on college campuses. The core modeling team facilitated each session and worked between sessions to ensure the successful development of the model. Specific activities included identifying and prioritizing the causes and consequences of alcohol-involved sexual violence, characterizing the causal relationships between these factors, and developing and modifying causal loop diagrams to illustrate these relationships.

**Results:**

Both students and practitioners identified key causes and consequences, including both individual-level (e.g., drinking to intoxication) and campus-level (e.g., institutional support for survivors) constructs. Both groups identified the causal relationships between these variables and identified salient, modifiable mechanisms for reducing alcohol-involved sexual violence.

**Conclusions:**

The collaborative model-building process successfully included diverse collaborator voices, integrating influential factors across multiple social-ecological levels. This iterative and capability-building approach can bridge intensive modeling efforts with the implementation and development of more effective sexual violence interventions.

Sexual violence—broadly defined to include sexual coercion, nonconsensual sexual contact, and rape (Basile et al., 2014)—is a persistent and significant public health issue on college campuses (Fedina et al., 2018). Although estimates vary due to variations in definitions of sexual violence and the specific population under study, approximately 1 in 5 women and 1 in 20 men experience sexual violence victimization during college (Fedina et al., 2018; Howard et al., 2019; Jouriles et al., 2022). The prevalence of lifetime sexual violence victimization is even higher among college students from minoritized backgrounds, including those who identify as sexual and gender minorities and students with disabilities (Chugani et al., 2022; Coulter et al., 2017).

Sexual violence perpetration (Abbey, 2011) and victimization (Testa & Livingston, 2018) are both associated with event-level alcohol consumption (Pedersen et al., 2022) as well as overall alcohol consumption patterns (Basile et al., 2021; Bryan et al., 2016). Multilevel factors—operating at individual, interpersonal, campus, and societal levels—along with their interactions, collectively contribute to sexual violence. For instance, campus-level social settings like fraternity/sorority parties, which are popular among students interested in drinking and impersonal sex, amplify both hazardous drinking and sexual violence and are linked to subsequent sexual aggression perpetration (Cleveland et al., 2019). These campus settings also reduce the likelihood of bystander intervention behaviors at the interpersonal level (Bannon et al., 2013; Canan et al., 2018).

Despite increased attention to preventing campus sexual violence over the past decade, its prevalence has not decreased (Fedina et al., 2018; Muehlenhard et al., 2017; Steele et al., 2024). Although effective prevention programs should address multiple levels of influence and their interactions (Dills et al., 2016), many programs focus solely on individual and interpersonal behaviors (Bonar et al., 2022), neglecting college- and community-level environments. Furthermore, a lack of diverse perspectives (in terms of personal identities and formal and informal campus roles) on the key causes and consequences of sexual violence on college campuses is another reason we have a relatively narrow set of prevention approaches (McMahon et al., 2021). Developing a better mechanistic and multilevel understanding of sexual violence on college campuses can help us evaluate and implement existing interventions, as well as develop new ones (Burke et al., 2022).

Using systems science to examine sexual violence on college campuses is a promising approach for advancing our understanding of this complex issue. Systems are composed of multilevel factors that interact over time, producing emergent properties not explained by any single element (Burke et al., 2020; Mabry et al., 2008). Systems science methods have been successfully applied to a range of health behaviors to improve understanding of complex problems (Apostolopoulos et al., 2018; El-Bassel et al., 2021; Mills et al., 2023; Savona et al., 2023). Collaborative and participatory systems science methods, in particular, can help us better understand the dynamics of alcohol-involved sexual violence on college campuses. These methods facilitate the development of shared mental models and support transdisciplinary learning and collaboration (Frerichs et al., 2016). Collaborative model-building (CMB), based on the group model-building approach (Allender et al., 2015; Andersen et al., 1997; Hovmand, 2014; Moore et al., 2021), is a systems science method for collectively constructing a model that illustrates the connections between the causes and consequences of complex issues (Gerritsen et al., 2020). This process draws on diverse perspectives and knowledge to identify key causes and consequences, incorporating both scientific evidence and lived experiences, which enhances the relevance and utility of the models. These participatory systems science approaches have been successfully used to develop models of the causes of cardiovascular risk disparities (Bauer et al., 2023), diet and food access disparities (Langellier et al., 2024), and overdose bystander behavior and Good Samaritan Laws (Thompson et al., 2024), among other pressing public health concerns.

In this article, we demonstrate how CMB, a participatory systems science method, can be applied to the study of alcohol-involved sexual violence. We walk through each step of the process, showing how it engages diverse collaborators to identify multilevel influences and consequences and how it reveals feedback loops that shape the prevalence of alcohol-involved sexual violence. We aim to show how systems-based methodologies can improve the development and implementation of interventions by addressing the complex dynamics that sustain alcohol-involved sexual violence. We also emphasize the unique and complementary perspectives that diverse collaborators bring to this complex problem.

## Method

### Overview

As part of the Collaborative Model Building Project to Understand Sexual Violence (CAMPUS), we collaboratively developed a systems model of alcohol use and sexual violence on college campuses. The main goal of the CMB activities was to collaboratively characterize the systems of alcohol use and sexual violence on college campuses. CMB activities were conducted separately with two cohorts (students and practitioners). The CMB activities were determined not to constitute human subjects research by the University of Pittsburgh Institutional Review Board (IRB). This determination was based on the nature of the activities, which focused on the development of systems models through the aggregation of participants' knowledge and perspectives rather than the collection of individual-level data for research purposes. The IRB's assessment ensured compliance with ethical standards and guidelines, affirming that the CMB process involved collaborative, participatory methods that did not directly involve human subjects research protocols.

### Collaborators

CMB activities were conducted with a group of 8 practitioners (e.g., campus health/sexual violence prevention educators) in April through June 2023, and with a group of 12 undergraduate students from various campuses in Allegheny County PA, from September through November 2023. We recruited collaborators through outreach to campuses in Allegheny County, PA, a county in Western Pennsylvania with more than eight undergraduate college campuses. Contacts at these institutions, involved in ongoing sexual violence prevention efforts (Abebe et al., 2018), distributed a flyer describing the study goal and a link to a Qualtrics screener survey. The survey assessed demographics, institutional affiliation, availability, professional role (for practitioners), and, for students, year of study, major, living situation (oncampus or off-campus), and extracurricular activities.

To ensure equity and representation, we purposively sampled practitioners and students to achieve maximum diversity across institutions, demographic characteristics (age, race/ethnicity, gender identity, and sexual orientation), professional roles (for practitioners), and student characteristics (e.g., year of study). Students enrolled only in online courses were excluded. Collaborators were compensated up to $375 for their participation in the CMB process.

### Process

With each cohort, we conducted a series of four in-person 2-hour CMB sessions, interspersed with work by the Core Modeling Team (CMT). The CMT consisted of seven faculty, postdoctoral fellows, and graduate students with expertise in alcohol, sexual violence, group model building, systems science, community-engaged research, and college health intervention implementation. Some of us identify as heterosexual cisgender men and women, some as cisgender gay and bisexual men; most of us identify as White, and some as Asian. Some of us have experienced sexual violence ourselves or through close friends and family. Each session was attended by at least three facilitators from the CMT. Sessions occurred 1–3 weeks apart, depending on the amount of work needed by the CMT in preparation for the next session. During or in between each session, the CMT members followed a process in which we listened carefully to one another; checked all assumptions with the peer-reviewed literature, other non-CMT experts, and our collaborators; and carefully documented all processes and decisions. Table 1 outlines activities undertaken in each of the four sessions, as well as CMT activities between sessions. All sessions were audio recorded, and photos were taken to document the process and session outputs (e.g., connection circles drawn on whiteboards in Session 2, described below).

**Table 1.**
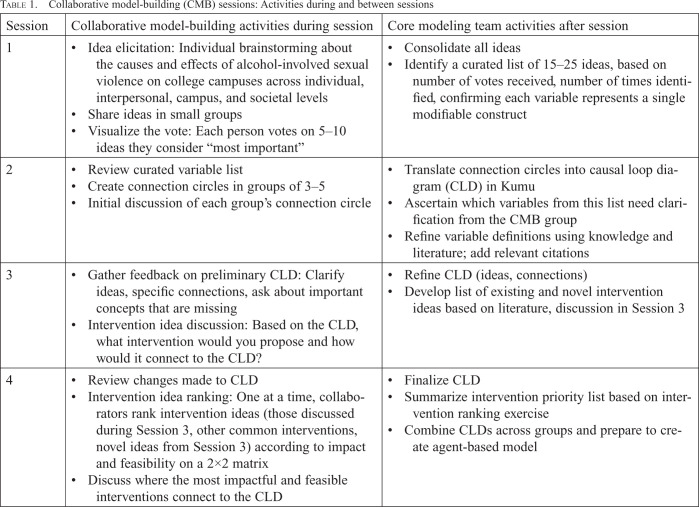
Collaborative model-building (CMB) sessions: Activities during and between sessions

Session	Collaborative model-building activities during session	Core modeling team activities after session
1	Idea elicitation: Individual brainstorming about the causes and effects of alcohol-involved sexual violence on college campuses across individual, interpersonal, campus, and societal levels Share ideas in small groups Visualize the vote: Each person votes on 5–10 ideas they consider “most important”	Consolidate all ideas Identify a curated list of 15–25 ideas, based on number of votes received, number of times identified, confirming each variable represents a single modifiable construct
2	Review curated variable list Create connection circles in groups of 3–5 Initial discussion of each group's connection circle	Translate connection circles into causal loop diagram (CLD) in Kumu Ascertain which variables from this list need clarification from the CMB group Refine variable definitions using knowledge and literature; add relevant citations
3	Gather feedback on preliminary CLD: Clarify ideas, specific connections, ask about important concepts that are missing Intervention idea discussion: Based on the CLD, what intervention would you propose and how would it connect to the CLD?	Refine CLD (ideas, connections) Develop list of existing and novel intervention ideas based on literature, discussion in Session 3
4	Review changes made to CLD Intervention idea ranking: One at a time, collaborators rank intervention ideas (those discussed during Session 3, other common interventions, novel ideas from Session 3) according to impact and feasibility on a 2×2 matrix Discuss where the most impactful and feasible interventions connect to the CLD	Finalize CLD Summarize intervention priority list based on intervention ranking exercise Combine CLDs across groups and prepare to create agent-based model

In Session 1, the primary goals were to introduce all collaborators to CMB, identify and share ideas about the causes and consequences of alcohol-involved sexual violence, and vote on the most important brainstormed ideas (variables). Collaborators were prompted to consider ideas at the individual, interpersonal, campus, and societal levels, writing each idea on a separate sticky note. They shared their ideas in small groups, reported to the entire group, and voted on the most important ideas by placing stickers on the sticky notes.

After Session 1, the CMT transcribed and synthesized the variables, tabulated the number of votes for each, and produced a curated list of 15–25 ideas. This list was based on the number of votes an idea received, the frequency with which it was identified, and ensuring that each variable represented a distinct, modifiable construct. Each included variable was given a brief definition, and the remaining clarifying questions were identified for discussion in Session 2.

During Session 2, facilitators presented the curated variable list to collaborators and discussed outstanding questions, which typically focused on establishing a shared understanding of variable definitions (e.g., Group 1 [practitioners] facilitators asked for clarification on the meaning of “campus party culture,” which was later split up into multiple variables). Collaborators then broke into smaller groups of three to five to create connection circles using whiteboards, sticky notes, and markers. Connection circles are graphical representations of the causes and consequences of a given problem, where the causal relationships between variables are identified, enabling the identification of feedback loops (Quaden et al., 2008). Collaborators used the connection circles to draw arrows between variables from the curated list, representing the causal relationships between the causes and consequences of sexual violence.

After Session 2, the CMT translated the connection circles into one unified causal loop diagram (CLD) by transferring the variables and the arrows drawn by participants into Kumu (Kumo Inc., Los Gatos, CA), a freely available online systems mapping platform combining like arrows and identifying conflicting arrows for further discussion with the CMT. CLDs serve as tools for visualizing relationships and changes over time (Williams & Hummelbrunner, 2010) and consist of variables (“nodes”), links between variables (“edges”), and signs on each edge that indicate whether the nodes change in the same direction (“positive edges”) or opposite direction (“negative edges”; Baugh Littlejohns et al., 2021; Smeekes et al., 2023; Vermaak, 2016). Each edge is directed, with a start and end of the edge indicated by the arrow pointing toward the end. So, a positive edge from “drinking to intoxication” to “alcohol-involved sexual violence” indicates that when drinking to intoxication increases, sexual violence also increases. Arrows can be bidirectional if two variables cause each other to increase/decrease. CLDs reveal a causal network of interconnected feedback loops, replacing linear cause-and-effect relationships that are inadequate for complex systems. Closed loops in CLDs are crucial as they represent sequences of causes and effects, illustrating the overall dynamics within the system. A single CLD can contain dozens of nodes, edges, and loops, highlighting key variables, their interconnections, and whether their interactions have reinforcing or dampening effects. After the initial translation, the CMT met several times to refine the CLD, variable names, and definitions using published literature, transcribed notes from Sessions 1 and 2, and their expertise in alcohol-involved sexual violence.

In Session 3, facilitators presented the preliminary CLD to each group and elicited collaborator feedback on specific variables and connections between variables flagged by the CMT as needing clarification (e.g., facilitators asked Group 1 whether there were missing connections associated with “likelihood of bystander intervention”). Collaborators also provided input on whether important variables/ideas were missing from the CLD (e.g., Group 2 [students] discussed how alcohol-related variables were missing and talked about which variables to add and how). Also in Session 3, collaborators brainstormed and discussed answers to the question, “Based on our discussion of the CLD so far, what intervention would you propose and how would it connect to the CLD?” After Session 3, the CMT refined the CLD based on collaborators' feedback (e.g., by adding variables or connections that were missing).

In Session 4, facilitators reviewed changes made by the CMT to the CLD. Finally, using a curated list of existing evidence-based interventions and intervention ideas generated in Session 3 (Supplemental Table 1), collaborators ranked each idea on a 2 × 2 matrix by both potential impact (*x*-axis) and feasibility of implementation (*y*-axis). (Supplemental material appears as an online-only addendum to this article on the journal's website.) One at a time, collaborators took an intervention written on a sticky note and placed it on the matrix (drawn on a whiteboard) until all interventions were added. To wrap up Session 4, collaborators discussed where those interventions identified as most impactful and feasible would connect to the CLD and how they would influence the outcome of interest. This discussion was used to help us understand salient mechanisms within the CLD. For example, Group 1 discussed how peer mentorship during freshman orientation could increase students' sense of belonging, increasing the likelihood of bystander intervention and decreasing the number of cases of sexual violence.

After the final session, the CMT continued to finalize each CLD and began the process of combing CLDs across groups in preparation for eventual translation into an agent-based modeling framework (Grefenstette et al., 2013; Koh et al., 2018).

### Products

**Figure 1.**
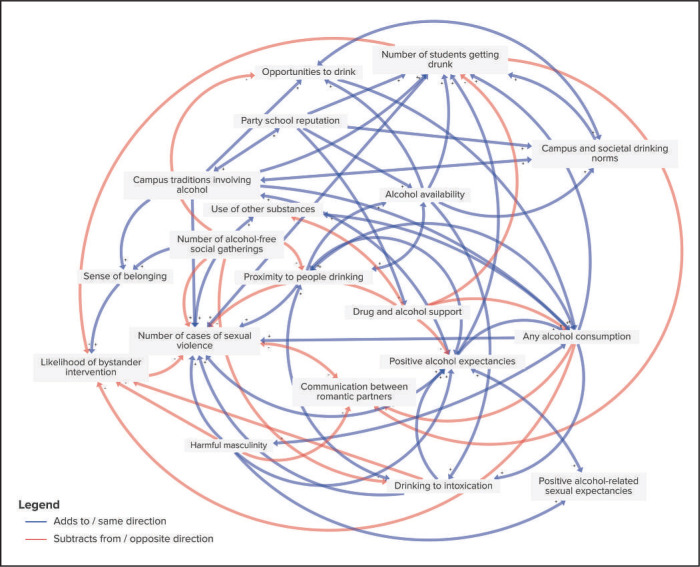
Final causal loop diagram created by practitioners (*n* = 8)

**Figure 2.**
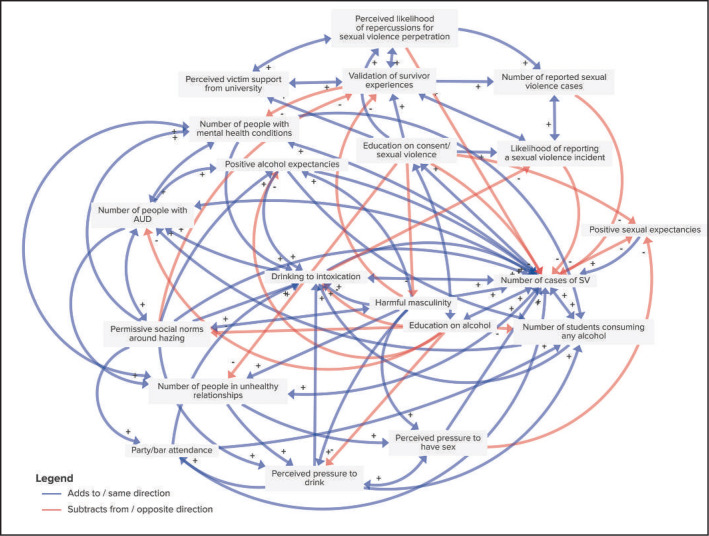
Final causal loop diagram created by students (*n* = 12)

For each cohort, the final output was a CLD of the causes and consequences of alcohol-involved sexual violence on college campuses (Figures 1 and 2) and a list of existing and novel intervention ideas ranked by impact and feasibility. In this article, we compare the lists of causes and consequences of sexual violence generated by each group, looking for similarities and differences, as well as the final CLDs. We have not included the discussion of interventions or the importance/feasibility matrix results.

## Results

We engaged collaborators with diverse backgrounds, perspectives, and roles. Table 2 contains details about the backgrounds of practitioner and student collaborators and the characteristics of the campuses they were affiliated with. Collaborators came from five colleges/universities in Allegheny County, including a community college, a small urban private university, a private suburban Catholic university, a private research university, and a large public R1 university. Collaborators had a diverse set of racial and gender identities and sexual orientations. The practitioners held roles in diversity and inclusion, sexual violence prevention education, residence life, Title IX coordination, and campus counseling. Students spanned all four undergraduate years, lived both on and off campus, and participated in extracurricular activities including fraternity/sorority life, college athletics, residence life, and student government.

**Table 2.**
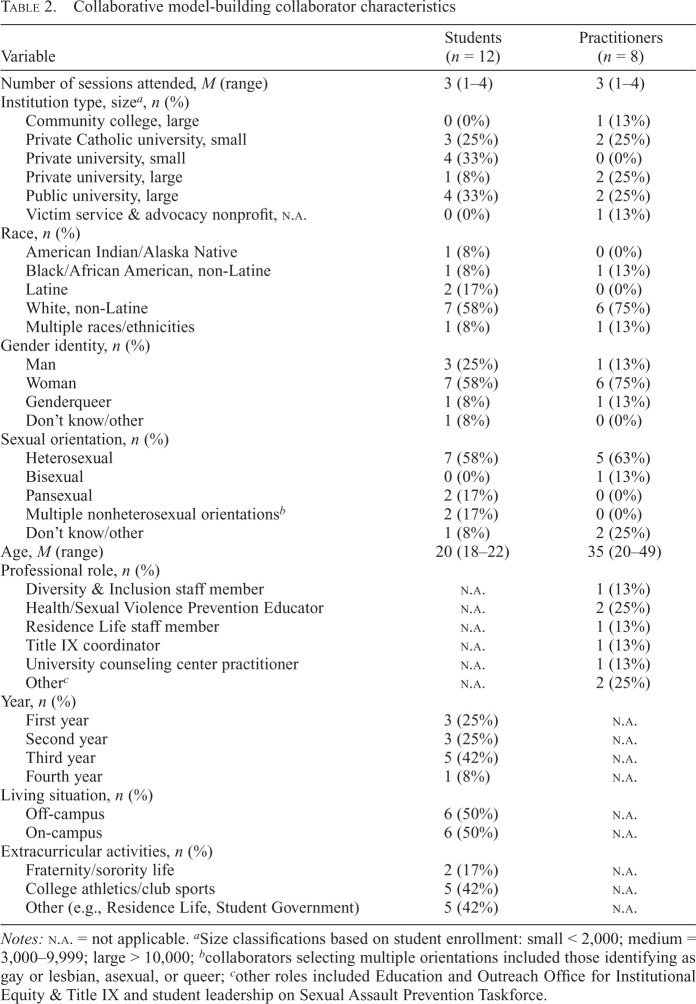
Collaborative model-building collaborator characteristics

Variable	Students (*n* = 12)	Practitioners (*n* = 8)
Number of sessions attended, *M* (range)	3 (1–4)	3 (1–4)
Institution type, size*^a^, n* (%)		
Community college, large	0 (0%)	1 (13%)
Private Catholic university, small	3 (25%)	2 (25%)
Private university, small	4 (33%)	0 (0%)
Private university, large	1 (8%)	2 (25%)
Public university, large	4 (33%)	2 (25%)
Victim service & advocacy nonprofit, n.a.	0 (0%)	1 (13%)
Race, *n* (%)		
American Indian/Alaska Native	1 (8%)	0 (0%)
Black/African American, non-Latine	1 (8%)	1 (13%)
Latine	2 (17%)	0 (0%)
White, non-Latine	7 (58%)	6 (75%)
Multiple races/ethnicities	1 (8%)	1 (13%)
Gender identity, *n* (%)		
Man	3 (25%)	1 (13%)
Woman	7 (58%)	6 (75%)
Genderqueer	1 (8%)	1 (13%)
Don't know/other	1 (8%)	0 (0%)
Sexual orientation, *n* (%)		
Heterosexual	7 (58%)	5 (63%)
Bisexual	0 (0%)	1 (13%)
Pansexual	2 (17%)	0 (0%)
Multiple nonheterosexual orientations*^b^*	2 (17%)	0 (0%)
Don't know/other	1 (8%)	2 (25%)
Age, *M* (range)	20 (18–22)	35 (20–49)
Professional role, *n* (%)		
Diversity & Inclusion staff member	n.a.	1 (13%)
Health/Sexual Violence Prevention Educator	n.a.	2 (25%)
Residence Life staff member	n.a.	1 (13%)
Title IX coordinator	n.a.	1 (13%)
University counseling center practitioner	n.a.	1 (13%)
Other*^c^*	n.a.	2 (25%)
Year, *n* (%)		
First year	3 (25%)	n.a.
Second year	3 (25%)	n.a.
Third year	5 (42%)	n.a.
Fourth year	1 (8%)	n.a.
Living situation, *n* (%)		
Off-campus	6 (50%)	n.a.
On-campus	6 (50%)	n.a.
Extracurricular activities, *n* (%)		
Fraternity/sorority life	2 (17%)	n.a.
College athletics/club sports	5 (42%)	n.a.
Other (e.g., Residence Life, Student Government)	5 (42%)	n.a.

*Notes:* N.A. = not applicable.

^a^ Size classifications based on student enrollment: small < 2,000; medium = 3,000–9,999; large > 10,000;

^b^ collaborators selecting multiple orientations included those identifying as gay or lesbian, asexual, or queer;

^c^ other roles included Education and Outreach Office for Institutional Equity & Title IX and student leadership on Sexual Assault Prevention Taskforce.

In Session 1, Group 1 identified 73 unique variables representing the causes and effects of alcohol-involved sexual violence, and Group 2 identified 126 variables. Both groups listed variables across the social-ecological levels (individual, interpersonal, campus, and societal). The final curated lists of variables that were eventually included in connection circles and CLDs are listed in Table 3, along with the main outcome of interest for both CLDs, which we defined as the “number of cases of sexual violence.” The practitioners included 19 variables, and the students included 20 variables; 3 were included by both students and practitioners. Both groups identified harmful masculinity, drinking to intoxication, and positive alcohol expectancies as important factors related to sexual violence. Variables relating to alcohol consumption at the individual level (e.g., perceived pressure to drink, any alcohol consumption) and campus level (e.g., proximity to people drinking, number of students getting drunk) were included by both groups, although the specific variables were slightly different. Students focused on their own perceptions of safety and support for sexual violence victimization, such as perceived victim support from the college, perceived likelihood of repercussions for perpetration, validation of survivor experiences, and permissive social norms around hazing. They drew from recent first-hand observations, which often led to more specific observations than the practitioners, who tended to take a higher-level perspective. For example, practitioners identified “campus and societal drinking norms,” whereas students more specifically identified “permissive social norms around hazing.” Practitioners identified constructs around community connectedness, such as the number of alcohol-free social gatherings and the sense of belonging on a campus.

**Table 3.**
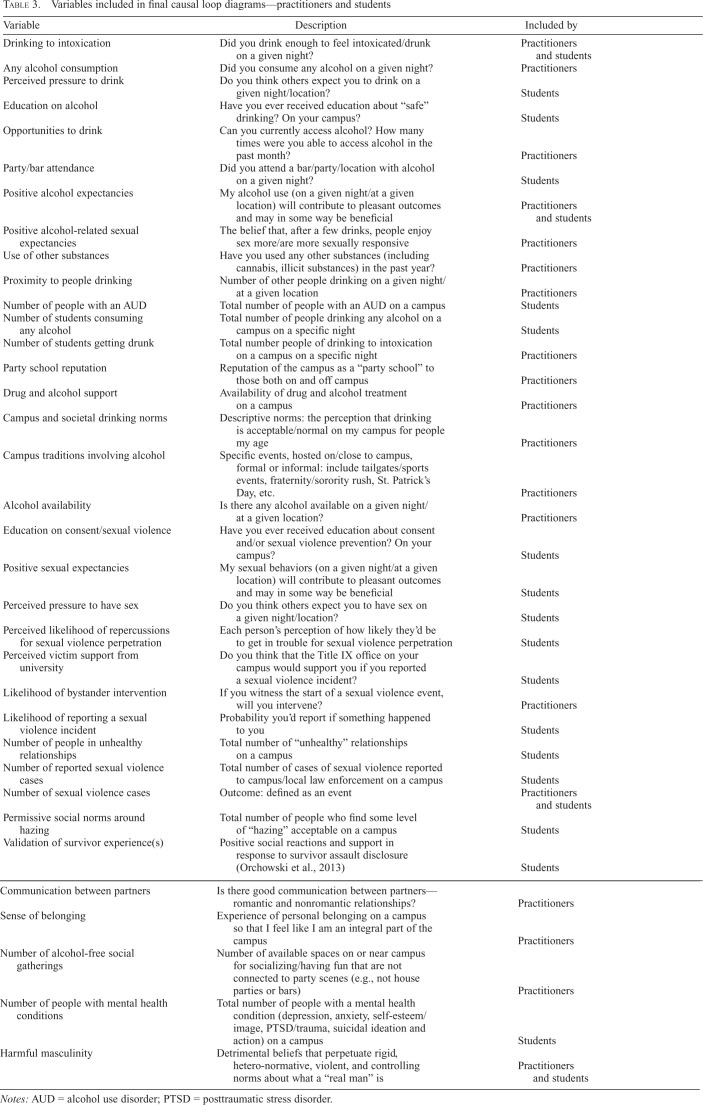
Variables included in final causal loop diagrams—practitioners and students

Variable	Description	Included by
Drinking to intoxication	Did you drink enough to feel intoxicated/drunk on a given night?	Practitioners and students
Any alcohol consumption	Did you consume any alcohol on a given night?	Practitioners
Perceived pressure to drink	Do you think others expect you to drink on a given night/location?	Students
Education on alcohol	Have you ever received education about “safe” drinking? On your campus?	Students
Opportunities to drink	Can you currently access alcohol? How many times were you able to access alcohol in the past month?	Practitioners
Party/bar attendance	Did you attend a bar/party/location with alcohol on a given night?	Students
Positive alcohol expectancies	My alcohol use (on a given night/at a given location) will contribute to pleasant outcomes and may in some way be beneficial	Practitioners and students
Positive alcohol-related sexual expectancies	The belief that, after a few drinks, people enjoy expectancies sex more/are more sexually responsive	Practitioners
Use of other substances	Have you used any other substances (including cannabis, illicit substances) in the past year?	Practitioners
Proximity to people drinking	Number of other people drinking on a given night/at a given location	Practitioners
Number of people with an AUD	Total number of people with an AUD on a campus	Students
Number of students consuming any alcohol	Total number of people drinking any alcohol on a campus on a specific night	Students
Number of students getting drunk	Total number people of drinking to intoxication on a campus on a specific night	Practitioners
Party school reputation	Reputation of the campus as a “party school” to those both on and off campus	Practitioners
Drug and alcohol support	Availability of drug and alcohol treatment on a campus	Practitioners
Campus and societal drinking norms	Descriptive norms: the perception that drinking is acceptable/normal on my campus for people my age	Practitioners
Campus traditions involving alcohol	Specific events, hosted on/close to campus, formal or informal: include tailgates/sports events, fraternity/sorority rush, St. Patrick's Day, etc.	Practitioners
Alcohol availability	Is there any alcohol available on a given night/at a given location?	Practitioners
Education on consent/sexual violence	Have you ever received education about consent and/or sexual violence prevention? On your campus?	Students
Positive sexual expectancies	My sexual behaviors (on a given night/at a given location) will contribute to pleasant outcomes and may in some way be beneficial	Students
Perceived pressure to have sex	Do you think others expect you to have sex on a given night/location?	Students
Perceived likelihood of repercussions for sexual violence perpetration	Each person's perception of how likely they'd be to get in trouble for sexual violence perpetration	Students
Perceived victim support from university	Do you think that the Title IX office on your campus would support you if you reported a sexual violence incident?	Students
Likelihood of bystander intervention	If you witness the start of a sexual violence event, will you intervene?	Practitioners
Likelihood of reporting a sexual violence incident	Probability you'd report if something happened to you	Students
Number of people in unhealthy relationships	Total number of “unhealthy” relationships on a campus	Students
Number of reported sexual violence cases	Total number of cases of sexual violence reported to campus/local law enforcement on a campus	Students
Number of sexual violence cases	Outcome: defined as an event	Practitioners and students
Permissive social norms around hazing	Total number of people who find some level of “hazing” acceptable on a campus	Students
Validation of survivor experience(s)	Positive social reactions and support in response to survivor assault disclosure (Orchowski et al., 2013)	Students
Communication between partners	Is there good communication between partners—romantic and nonromantic relationships?	Practitioners
Sense of belonging	Experience of personal belonging on a campus so that I feel like I am an integral part of the campus	Practitioners
Number of alcohol-free social gatherings	Number of available spaces on or near campus for socializing/having fun that are not connected to party scenes (e.g., not house parties or bars)	Practitioners
Number of people with mental health conditions	Total number of people with a mental health condition (depression, anxiety, self-esteem/image, PTSD/trauma, suicidal ideation and action) on a campus	Students
Harmful masculinity	Detrimental beliefs that perpetuate rigid, hetero-normative, violent, and controlling norms about what a “real man” is	Practitioners and students

*Notes:* AUD = alcohol use disorder; PTSD = posttraumatic stress disorder.

The analysis of the CLDs from Group 1 and Group 2 revealed several significant loops that underscore the complex interplay of factors contributing to alcohol-involved sexual violence on college campuses. Both groups highlighted key variables such as drinking to intoxication, harmful masculinity, positive alcohol expectancies, and the number of cases of sexual violence, which formed the core of the overlapping loops between the two diagrams. Still, significant differences were noted. Group 1 focused more on environmental and cultural factors, such as campus traditions and “party school” reputation, and their influence on alcohol consumption and sexual violence. In contrast, Group 2 placed a stronger emphasis on educational interventions and mental health, highlighting the importance of education on alcohol and consent, mental health conditions, and the perceived pressure to drink and have sex.

In Group 1, the main loops identified included one that illustrated how increased alcohol availability and campus traditions involving alcohol lead to higher alcohol consumption, subsequently increasing the number of sexual violence cases and reinforcing the party school reputation. A second loop showed how harmful masculinity reduces communication between romantic partners, increasing cases of sexual violence and alcohol consumption. A third loop emphasized the role of alcohol-free social gatherings in reducing the proximity to people drinking and potentially lowering the number of cases of sexual violence.

Group 2 identified a loop that highlighted how enhanced education on alcohol and consent/sexual violence increases the perceived likelihood of repercussions and validation of survivor experiences, leading to more reported cases of sexual violence. A second loop emphasized the contributions of mental health conditions to unhealthy relationships and increased sexual violence incidents. A third loop underscored the importance of perceived victim support and validation of survivor experiences in enhancing reporting and accountability, thereby reducing sexual violence.

The common loops across both diagrams emphasize the cyclical pattern in which increased drinking leads to more sexual violence, which in turn reinforces the drinking culture and harmful social norms. The loops also highlighted the potential impact of comprehensive interventions, including improved education, support services, and changing cultural norms, in addressing and preventing sexual violence on college campuses.

## Discussion

Our CMB process generated models of the causes and consequences of sexual violence relevant to collaborators with diverse perspectives. The two groups, students and practitioners, developed models that both overlapped and diverged. Many variables included in both models were supported by the existing evidence base while also involving more novel constructs, such as the availability of third spaces (i.e., places that are neither home [first space] nor work [second space], but rather serve as social gathering spots where people can relax, socialize, and engage in various activities) (Oldenburg, 1999; Williams & Dodge Francis, 2022) and permissive social norms around hazing. Below, we discuss specific ways our collaborative systems science approach highlighted distinct interconnected and multilevel influences and consequences of alcohol-involved sexual violence on college campuses.

Alcohol played a key role in both CLDs. This is important because alcohol remains an underdeveloped intervention lever to reduce sexual violence on college campuses, for reasons both compelling (e.g., fear of blaming victims) and related to the siloing of intervention efforts (Klein et al., 2021; Koss et al., 2022; Leone et al., 2022). Students and practitioners identified a range of variables related to alcohol consumption on college campuses, reflecting its multilevel importance. These included patterns of individual consumption (e.g., drinking to intoxication), aggregate behavioral patterns across a campus (e.g., number of students with an alcohol use disorder), contexts in which drinking occurs (e.g., party/bar attendance), and long-standing structural norms around alcohol consumption and the reputation of a campus (e.g., campus traditions involving alcohol). These variables had direct and indirect impacts on sexual violence and were present in most closed loops. Although alcohol consumption is modifiable, through both behavioral and structural interventions, both students and practitioners discussed that changing access to alcohol and reducing the central (social) importance of drinking would be extremely difficult. This highlights the lack of communication between alcohol and sexual violence practitioners, as many environmental alcohol prevention strategies have been successfully implemented and reduced alcohol-related problems (Ringwalt et al., 2011; Saltz et al., 2010). Such strategies include limiting alcohol promotions and sales in and around campuses, creating alcohol-free events, requiring keg registrations, and enforcing seller penalties for underage sales of alcohol (DeJong & Langford, 2002). Inclusive, participatory research methods can ensure that the breadth of relevant perspectives are integrated into intervention development and implementation.

Practitioners and students highlighted variables related to sexual behaviors, such as the need for more and better sex education, communication between partners, understanding of sexual expectancies, and the roles of sexuality and sex on campus and in society. They saw sexual violence as the end of the continuum of healthy-to-unhealthy sexual experiences and wanted campuses to better support healthy sexual relationships. Providing holistic sexual health resources to students is an important and underdeveloped avenue for reducing sexual violence (Eisenberg et al., 2012).

CMB activities enabled the co-production of CLDs between the CMT and collaborators, mitigating traditional power dynamics that often occur with other qualitative research methodologies (Freebairn et al., 2018; Gerritsen et al., 2020; Kaaristo, 2022). The iterative process between the four collaborator sessions and the CMT activities allowed us to construct final CLDs that reflected a wide range of perspectives, as we were able to check our assumptions with both collaborators and the extant research literature, reconciling our “scientific” understanding with lived experiences of students and practitioners. The different activities across sessions also provided opportunities for each collaborator to participate in a variety of ways, helping ensure that the perspectives of collaborators who might be hesitant to speak up were captured (Gerritsen et al., 2020; Savona et al., 2023). In the final session, students noted that participating in the CMB sessions made them feel like their viewpoints were being heard by those in positions to affect campus-level policies.

Our key findings reaffirm what theorists and practitioners have discussed, such as the roles of harmful masculinity, alcohol intoxication, and campus drinking culture in sexual violence. However, one of the crucial contributions of the systems science approach is the identification of reinforcing feedback loops that perpetuate sexual violence. For example, both students and practitioners noted how increased alcohol consumption leads to more sexual violence, which then reinforces harmful campus norms around drinking and masculinity. The collaborative aspect of the CMB allowed for a synthesis of diverse perspectives, showing the value of participatory approaches in drawing attention to nuanced, mutually reinforcing relationships grounded in lived experiences. Findings underscore the need for cross-cutting prevention approaches that directly address these intersections of drinking and sexual violence.

### Limitations

Given the complexity of alcohol-involved sexual violence on college campuses, it is unsurprising that our CMB approach was limited in several ways. Altogether, the two groups brainstormed hundreds of causes and consequences of sexual violence, which were winnowed down to much shorter lists for development into CLDs. We worked to ensure that the most common and potentially important ideas were included, and there remain many causal mechanisms not included in either CLD. We limited the focus of our model building to variables that are modifiable by colleges and universities, as the eventual end users of the agent-based model to be developed by this project are decision-makers on college campuses. Variables identified, such as the importance of early education on consent and sexual violence, are no longer modifiable once a person reaches college; there are also constructs, such as the ways that sexuality is portrayed in media, that are outside the scope of influence for college staff and administrators. The next step in our model development process will be to integrate a set of potential intervention levers. We did not ask our collaborators to build the CLDs explicitly with intervention opportunities in mind, meaning that some of the necessary closed loops for a given intervention are not in either CLD. The CMT will continue to modify and combine models with this in mind, and we plan to invite a smaller group of student and practitioner collaborators back to provide feedback throughout the next steps.

The eventual goal of CAMPUS is to build a quantitative systems model to be used in the development and implementation of campus-specific plans for reducing alcohol-involved sexual violence. The CMB process enabled us to pay crucial attention to diverse collaborator voices, receive feedback in an iterative manner, and integrate multiple levels of influential factors. We aim to build a bridge between intensive modeling efforts and implementation and development of more effective sexual violence interventions that center diverse perspectives and highlight potential resiliencies. In discussing connections and how interventions fit into the CLDs, it was evident that the CMB process facilitated mutual learning about sexual violence and available resources. For example, several student collaborators noted that this process helped them think more systemically about the causes and consequences of sexual violence and made them feel that they were being listened to and had valuable perspectives to contribute. They stated that they now had a much more comprehensive understanding of contributing factors and were eager to share their knowledge of potential resources with their classmates and friends. Other collaborators mentioned that it helped them build relationships across institutions with other collaborators doing similar work or having similar interests. Participatory systems science can help the sexual violence field more specifically articulate the interconnected and multilevel mechanisms of alcohol-involved sexual violence on college campuses, enabling the implementation and development of interventions that specifically address these mechanisms.
